# Oncolytic Adenoviruses Armed with Thymidine Kinase Can Be Traced by PET Imaging and Show Potent Antitumoural Effects by Ganciclovir Dosing

**DOI:** 10.1371/journal.pone.0026142

**Published:** 2011-10-18

**Authors:** Daniel Abate-Daga, Nuria Andreu, Juan Camacho-Sánchez, Ramon Alemany, Raúl Herance, Olga Millán, Cristina Fillat

**Affiliations:** 1 Institut D'Investigacions Biomèdiques August Pi i Sunyer-IDIBAPS, Barcelona, Spain; 2 Centre de Regulació Genòmica (CRG), UPF, Barcelona, Spain; 3 Centro de Investigación Biomédica en Red de Enfermedades Raras (CIBERER), Barcelona, Spain; 4 Gene and Viral Therapy Group, IDIBELL-Catalan Institute of Oncology (ICO), L'Hospitalet de Llobregat, Barcelona, Spain; 5 Institut d'Alta Tecnologia, PRBB Fundació Privada, Barcelona, Spain; University of Michigan School of Medicine, United States of America

## Abstract

Replication-competent adenoviruses armed with thymidine kinase (TK) combine the concepts of virotherapy and suicide gene therapy. Moreover TK-activity can be detected by noninvasive positron emission-computed tomography (PET) imaging, what could potentially facilitate virus monitoring *in vivo*. Here, we report the generation of a novel oncolytic adenovirus that incorporates the Tat8-TK gene under the control of the Major Late Promoter in a highly selective backbone thus providing selectivity by targeting the retinoblastoma pathway. The selective oncolytic TK virus, termed ICOVIR5-TK-L, showed reduced potency compared to a non-selective counterpart. However the combination of ICOVIR5-TK-L with ganciclovir (GCV) induced a potent antitumoural effect similar to that of wild type adenovirus in a preclinical model of pancreatic cancer. Although the treatment with GCV provoked a reduction in the viral yield, both *in vitro* and *in vivo*, a two-cycle treatment of virus and GCV resulted in an enhanced antitumoral response that correlated with high TK-activity, based on microPET measurements. Thus, TK-expressing oncolytic adenoviruses can be traced by PET imaging providing real time information on the activity of the virus and its antitumoral potency can be optimized by GCV dosing.

## Introduction

Arming tumor selective adenoviruses to express a transgene combines gene therapy and virotherapy and facilitates the incorporation of complementary or synergistic mechanisms operating in a unique virus. We have previously shown that the insertion of the modified form of the herpes simplex virus thymidine kinase gene, Tat8TK under the control of the major late promoter in the adenoviral genome AdRGDTat8-TK-L results in a highly potent replication competent adenovirus when combined with ganciclovir (GCV) [1]. Tat8TK is an engineered version of TK that incorporates an eight-amino acid domain derived from the human immunodeficiency virus Tat protein. We have previously shown that this fusion protein displays greater cytotoxicity than wild type TK probably due to protein-transduction capabilities conferred by the Tat8 domain [2].

The elements of success of a therapeutic virus are based both on the efficacy in reduction of tumor burden and its preferential activity on tumor cells. Thus, replication-selective control of adenoviruses is fundamental to provide safety to oncolytic virotherapy. Oncolytic adenoviruses that target the pRB pathway were initially developed by deletion of the pRB binding site of E1A, generating a mutant adenovirus unable to dissociate pRB from E2F in quiescent normal cells, limiting the transcriptional activation of *E2* viral genes by E2F, thus avoiding viral replication [3], [4]. Additional modifications such as the insertion of an RGD peptide in the HI loop and the introduction of an insulated E2F promoter controlling E1A resulted in the improved pRB based selective oncolytic adenovirus ICOVIR-5 [5].

The possibility to non-invasively monitor the tumor or non-tumor location of a replication competent virus dramatically improves the quality on the follow-up of a viral therapy and can provide real-time information on the activity of the injected virus. Oncolytic adenoviruses expressing the TK gene are susceptible of being traced by positron emission-computed tomography (PET) scans. A number of radioactive substrates that can be detected in PET, based on the capacity of the herpes simplex viral thymidine kinase to phosphorylate a variety of substrates that can not be phosphorylated by the mammalian thymidine kinase have been described [6]. With this technique there are several examples documenting TK expression in preclinical models both in tumors [7], [8], [9] or in normal tissues such as in mouse or non-human primates liver upon intravenous (i.v.) adenoviral delivery [10], [11]. Importantly, TK expression has also been monitored by PET scans in patients with hepatocellular carcinoma upon injection with a first-generation adenovirus encoding the TK gene [12].

In this study we have developed a novel oncolytic adenovirus that incorporate the optimized Tat8-TK gene controlled by the adenoviral major late promoter in an ICOVIR-5 backbone and show that the potency of the selective virus, ICOVIR5-TK-L, can be significantly improved when combined with GCV in pancreatic tumors. Importantly we show that oncolytic adenoviruses expressing the TK gene can be monitored by PET and that the protocol of GCV administration impacts on the TK activity induced by ICOVIR5-TK-L.

## Materials and Methods

### Cell Lines

The human pancreatic adenocarcinoma cell lines BxPC-3, RWP1, PANC-1, NP-9 and NP-18, the A549 human lung cancer cells; and the HEK293 cells were obtained from the ATCC (Manasas, Virginia, United States) or from G. Capellà and FX. Real Laboratories [13], [14] and cultured as previously described [15].

### Adenovirus construction and infection

Construction of recombinant AdTK, AdTat8-TK and replication competent Adwt-RGD, AdRGDTat8-TK-L and ICOVIR-5 have been previously described [1], [16], [17], [18], [19]. Here we have generated a novel virus named ICOVIR5-TK-L. The plasmid pNKFiber RGDTat8TK [1] was digested with *Not*I and *Kpn*I and recombined by homologous recombination in yeast with the plasmid pICOVIR5SwaCAU. This plasmid derives from pICOVIR5 [5] by first inserting yeast replication elements in the plasmid backbone (a centromer CEN6 region modified to eliminate the *Swa*I site, an autonomous replicating region, and a Ura3 gene for selection) and later inserting a *Swa*I site in the adenovirus fiber to become a unique site for cut-repair homologous recombination in yeast. The recombination of the RGDTat8TK fiber in this plasmid resulted in pICOVIR5-TK-L. Confirmation of pICOVIR5-TK-L integrity was performed by analysing the presence of the different DNA elements (E2F promoter, E1A-Δ24 deletion, TK and hexon) by PCR and DNA sequencing. pICOVIR5-TK-L was cut with *Pac*I and transfected in HEK293 cells to generate ICOVIR5-TK-L which was plaque-isolated and propagated in A549 cells.

All viruses were purified by standard Caesium Chloride banding. The physical particle concentration (vp/ml) was determined by Optical Density reading (OD_260_) and the plaque forming units (pfu/ml) were determined on HEK293 cells by the anti-hexon staining-based method [1].

### RT-PCR analysis

RNA was extracted from a panel of cell lines infected with ICOVIR5-TK-L using TriPure isolation Reagent (Roche Applied Science, Sant Cugat del Vallès, Spain). To avoid genomic contamination RNA samples were treated with DNase (DNA-free, Ambion, Applied Biosystems, Madrid, Spain) as described by manufacturer's protocol. One µg of total RNA from each sample was reverse transcribed with a Retroscript RT kit (Ambion). Two µl of cDNA was PCR amplified with four pairs of specific primers for four different regions: promoter region (E2F-F: 5′ CGCGTTAAAGCCAATAGGAA 3′ and E1A-R2 5′ CGGCCATTTCTTCGGTAATA 3′), TK (TK5′: CTCATCCCGCCGACCT and TK3′: CACGACCCGCCGCCCTG), E1A (E1A5′: ATCGAAGAGGTACTGGCTGA and E1A3′: CCTCCGGTGATAATGACAAG) and hexon protein (hexon5′: GCCGCAGTGGTCTTACATGCACATC and hexon3′: CAGCACGCCGCGGATGTCAAAG).

### Determination of viral replication *in vitro*

RWP-1, NP-18 and BxPC-3 cells were seeded at 3×10^4^ cells per well in quadruplicate in 24-well plates and cultured overnight. The next day, cells were infected with Adwt-RGD, AdRGDTat8-TK-L or ICOVIR5-TK-L at 1×10^4^ vp per cell. Four hours post-infection, the medium was replaced by complete fresh medium. Twenty-four hours later the cells were incubated, either in the presence or absence of 10 µg/ml GCV, for 3 days. Then the cells and supernatants were collected and subjected to three cycles of freeze and thawing. A549 cells were infected for 24 h, with serial dilutions of total extracts, and the number of viral infective particles was quantifed by means of the hexon protein-staining technique.

### Dose-response analysis and ID_50_ values

NP-18, RWP-1 and BxPC-3 cells were seeded in 96-well plates at a density of 3×10^3^ cells per well in 96-well plates and cultured overnight. Twenty-four hours later, the cells were infected with Adwt-RGD, AdRGDTat8-TK-L or ICOVIR5-TK-L, using a dose range of 0 to 10^6^ vp/cell, in triplicates. Four hours post-infection the viral medium was replaced by a complete fresh medium. Twenty-four hours later the cells were incubated, either in the presence or absence of GCV, for 3 days. Cell viability was measured using the MTT colorimetric assay and ID_50_ values were estimated from dose-response curves by standard non-linear regression, using an adapted Hill Equation [15].

### Tumour growth studies

BxPC-3 cells (2.5×10^6^) were injected s.c. into each posterior flank of BALB/c nude mice (Charles River France, Lyon, France). Tumours were measured three times per week and volumes were calculated according to the formula V (mm^3^) = larger diameter (mm)×smaller diameter 2 (mm^2^)/2. Treatment was initiated when tumours achieved a mean volume of 50 mm^3^. All animal procedures met the guidelines of European Community Directive 86/609/EEC, and of the Local Catalan regulations (Law 5/1995, Decrees 214/97, 32/2007) and were approved by the Local Animal Care and Ethics Committee (CEEA-PRBB).

### Determination of viral yield in tumors

Viral genomes were determined in tumor samples by a quantitative PCR (qPCR) assay and SYBR^©^ Green chemistry (Roche). DNA was isolated using a DNeasy Blood and Tissue Kit (QIAGEN, Barcelona, Spain) according to the manufacturer's instructions. DNA concentration was determined spectrophotometrically and adjusted to 10 ng/µl, 5 µl of DNA was used for qPCR. A plasmid DNA ICOVIR5-TK-L was serially diluted (10–10^6^ copies, in a background of tumor genomic DNA) and used as a quantification standard for the real time qPCR. A conserved sequence of the adenovirus hexon gene was PCR amplified using forward and reverse hexon primers described above. Samples and standards were amplified in triplicate. Data were collected and analyzed using a LightCycler® 480 Real-Time PCR System (Roche Diagnostics, Mannheim, Germany). The lower limit of the linear range of the quantitative assay was determined to be 1000 adenovirus DNA copies in the background of tumor genomic DNA. Results are expressed as viral genomes in one µg of tumor DNA.

Viral infective particles in tumor samples were analyzed in homogenates prepared in isotonic buffer, and subjected to three cycles of freezing and thawing. Extracts were applied to A549 cells and the number of infectious particles were quantified by hexon staining.

### Radiolabeled substrate synthesis

All chemistry procedures were performed with HPLC grade solvents obtained from Scharlab (Barcelona, Spain), Panreac (Castellar del Vallès, Spain) or Sigma-Aldrich (Madrid, Spain). All chemicals were used without further purification and purchased from Sigma-Aldrich, Fluka (Sigma-Aldrich, Madrid, Spain) and Acros Organics (Thermo Fisher Scientific, Geel, Belgium). QMA and Alummina Sep-pack were obtained from Waters (Milford, MA), 0.22 µm filters from Millipore (Madrid, Spain) and 18-O enriched water from Marshall Isotopes. An 18/9 IBA (Ion Beam Application, Belgium) Cyclotron was used for [^18^F^−^]fluoride production and an IBA FDG Synthesizer Module was used for fluorination. Radioactivity was determined using a calibrated chamber (Comecer, Pet Dose) HPLC for [^18^F]FEAU purification was carried out on a modular Agilent 1100 series HPLC system with a semi-preparative reverse-phase C18 column (Agilent SB-18, 250×10 mm, 5 µm) equipped with UV (λ = 254) and radioactive detector (Raytest, Gita). For quality control, HPLC analysis was carried out on a modular Agilent 1100 series system with a reverse-phase C8 column (Agilent XDB-C8, 150×4.6 mm, 5 µm) equipped with diode-array UV (λ = 220 and 254) and radioactive detector (Raytest, Gita).

Non-radioactive standard and precursor were synthesized following published methodologies [20], [21], [22]. Full characterization of both compounds was performed using MS and ^1^H-NMR. [^18^F]FEAU was obtained following published methodologies with minor modifications [23], [24]. Briefly, [^18^F^−^]fluoride was obtained with 16 MeV protons irradiation of 18-O enriched water using the nuclear reaction ^18^O(p,n)^18^F, and trapped on a QMA cartridge. [^18^F]fluoride was then eluted using a solution of 28 mg Kryptofix 2.2.2 and 5 mg K_2_CO_3_ in a mixture of 700 µl of acetonitrile and 200 µl of water. The [^18^F^−^]fluoride solution was evaporated azeotropically at 100°C using an helium stream and vacuum three times with 1 ml of dry acetonitrile each. To the dry residue, a solution of 6 mg of precursor (2-O-(trifluoromethylsulfonyl)-1,3,5-tri-O-benzoyl-α-D-ribofuranose) in dry acetonitrile was added, and the reaction mixture was heated at 80°C for 20 min. The reaction mixture was cooled, passed through an alumina cartridge, and eluted with 2.5 ml of ethyl acetate. The solvent was removed applying a stream of nitrogen at 80°C. The residue was dissolved in 0.4 ml dichloroethane under inert helium atmosphere and 100 µl of hydrogen bromide in acetic acid (30%) was added. The reaction mixture was heated at 80°C for 10 min and evaporated using a nitrogen stream at 50°C. The dry residue was mixed with a fresh solution of 2,4-bis-O-(trimethylsilyl)thymidine (80 µmol in 0.5 ml dichloroethane). The mixture was then heated at 100°C for 60 min, cooled, passed through an alumina cartridge and eluted with 2.5 ml of 10% of methanol in dichloromethane. The solvent was evaporated using a nitrogen stream at 100°C. Then, the residue was dissolved in 300 µl methanol and 30 µl of sodium methoxide 1 M in methanol were added. This mixture was heated at 80°C during 5 min. The reaction crude was cooled at room temperature and HCl 2N was used. After neutralization the solvent was evaporated. The residue was solved in a 10∶90 mixture of acetonitrile/water and purified by sempreparative HPLC at a flow of 4 ml/min. The appropriate fraction eluted at about 20 min. After collection, it was evaporated until dryness, dissolved in 0.9% saline solution and passed trough a 0.22 µm filter. An aliquot of the final product was analyzed by analytical HPLC using a mixture of acetonitrile/water 10∶90 at a flow of 2 ml/min and identified by co-elution of standard. [^18^F]FEAU was obtained in 14±5% (n = 5) decay corrected yield with a chemical and radiochemical purity >97%.

#### *In vitro* ^18^F-FEAU uptake assay

To measure *in vitro* the TK activity of adenovirus-transduced tumor cells after the different treatments tested, an ^18^F-FEAU uptake assay was performed in duplicates. The same amount of radioactivity (35 KBq aprox.) was added to each well and cells were incubated at 37°C, 5% CO_2_ for 2 h. After this period, supernatants were removed and placed in a series of tubes. Cells were washed once with fresh medium, which, after removal, was transferred to a new series of tubes. Cells were then trypsinized and centrifuged at 1200 rpm for 5 min. The radioactivity in each of the three series of tubes was measured in a Wallac 1470 Wizard gamma counter (PerkinElmer, Waltham, MA). HSV1-TK activity was determined as a function of ^18^F-FEAU uptake by referring the count values obtained from each cellular pellet to those of a known standard.

#### MicroPET imaging

Mice bearing ICOVIR5TK-L-injected xenografts in the anterior flanks were imaged for TK activity using a microPET R4 (Concorde Microsystems, Knoxville, TN) and ^18^F-FEAU as a substrate for HSV1-TK enzyme. All animals were fed *ad libitum* and had continuous access to water up to the time of experiment. Mice were anesthetized with 1.5–2% isoflurane (Isoflo, Esteve) in oxygen and injected via tail vein with 2–4 MBq of ^18^F-FEAU two hours prior to a 12.5 min image acquisition. Imaging was performed on a prone position using an energy window of 350–650 keV and a coincidence timing window of 6 nsec. The resulting list-mode data were sorted into 2D histograms by Fourier re-binning and the images reconstructed by filtered back-projection into a 128×128×63 (0.82×0.82×1.3-mm) matrix. The image data were corrected for non-uniformity of response of the microPET, deadtime count losses, and physical decay to the time of injection but no attenuation, scatter, or partial-volume averaging correction was applied. Using a mouse-size phantom, an empirically determined system calibration factor (i.e. mCi/ml/cps/voxel) was used to convert voxel count rates to activity concentrations. The resulting image data were processed according to the software provided by the manufacturer and quantification was obtained by region-of interest analysis (ASIPro, Concorde Microsystems, Knoxville, TN).

### Statistical Analysis

The descriptive statistical analysis was performed on SPSS software (SYSTAT software, Inc, Chicago, IL). Results are expressed as mean ± SEM. A Mann-Whitney non-parametric test was used for the statistical analysis (2-tailed) of *in vitro* and *in vivo* studies. P<0.05 was taken as the level of significance.

Statistical analysis of *in vivo* tumour growth was performed by ANOVA for repeated measures, employing the SPSS software.

Survival analyses were also performed to analyze time-to-event probability using the SPSS software. The survival curves obtained were compared for the different treatments. A log-rank test was used to determine the statistical significance of the differences in time-to-event. A *p* value of less than 0.05 was considered statistically significant.

## Results

### GCV enhances the potency of ICOVIR5-TK-L in spite of interference with viral replication

**Figure 1:**
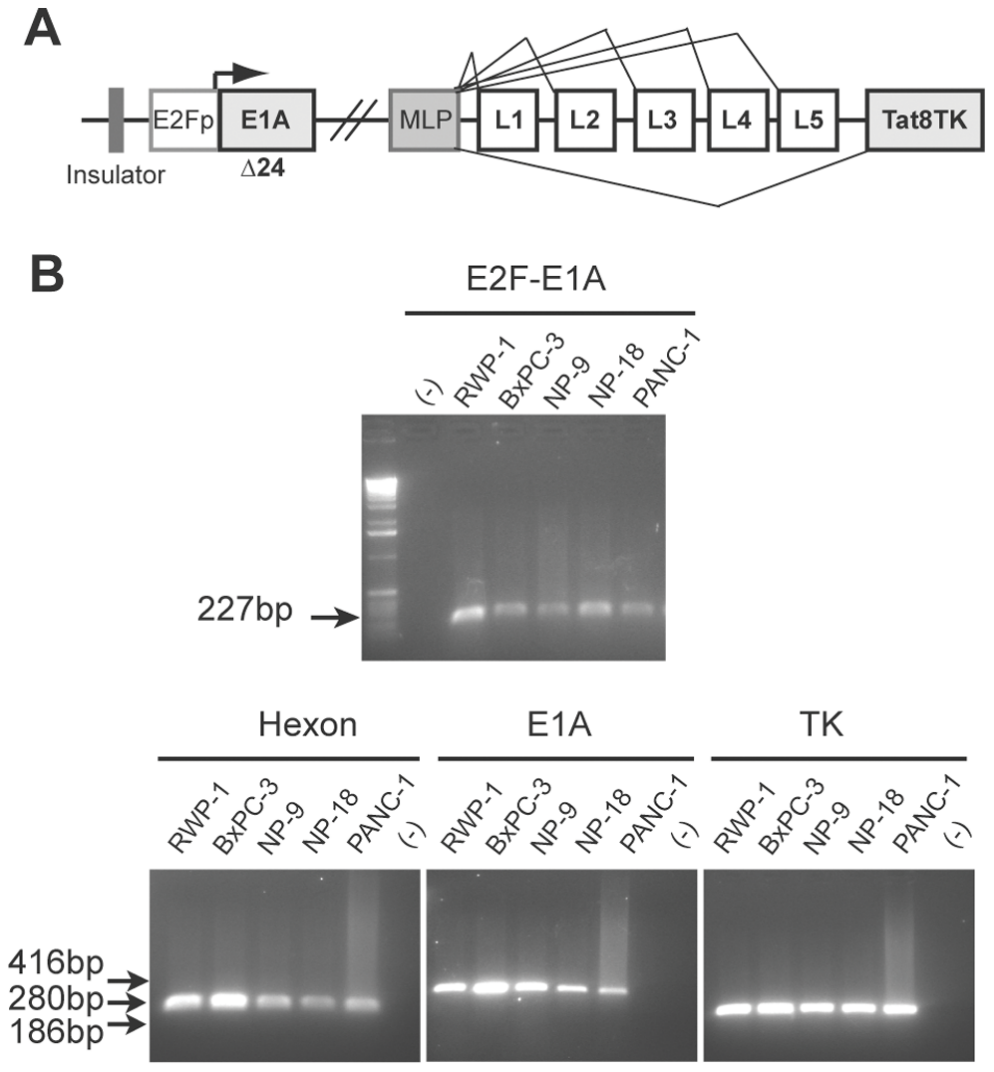
ICOVIR5-TK-L. (A) Schematic representation of adenoviral genomic elements. The E1A-Δ24 gene was cloned under the control of the E2F-1 promoter, preceded by an insulator sequence in the 5′ region. The Tat8TK transgene was cloned downstream of L5 to be expressed as a part of a polycystronic transcript, under the control of the Major Late Promoter. (B) Quality control assessment. The presence of E2F-1 promoter, and of Hexon, E1A and Tat8TK genes was analyzed by RT-PCR in pancreatic cancer cells transduced with 1000 vp/cell of ICOVIR5-TK-L. A non-retrotranscribed mRNA sample was used as a negative control for the PCR.

ICOVIR5-TK-L (Figure 1A) contains the Tat8-TK gene under the control of the adenoviral Major Late Promoter and is expressed as a splice variant from the IIIa splicing factor sequence identically to AdRGD-Tat8-TK-L [1]. It differs from the replicative competent AdRGD-Tat8-TK-L by the incorporation of all the selective control elements from ICOVIR-5 [5].

To confirm the integrity of the virus, a panel of pancreatic cancer cell lines were infected with ICOVIR5-TK-L at 1000 vp/cell and further analyzed for expression of viral elements. mRNA from cell lysates were extracted at 24 h post-infection and bands corresponding to E2F-E1A junction fragment, E1A, hexon and Tat8-TK mRNAs were obtained after RT-PCR analysis in every cell line analyzed (Figure 1B).

**Figure 2:**
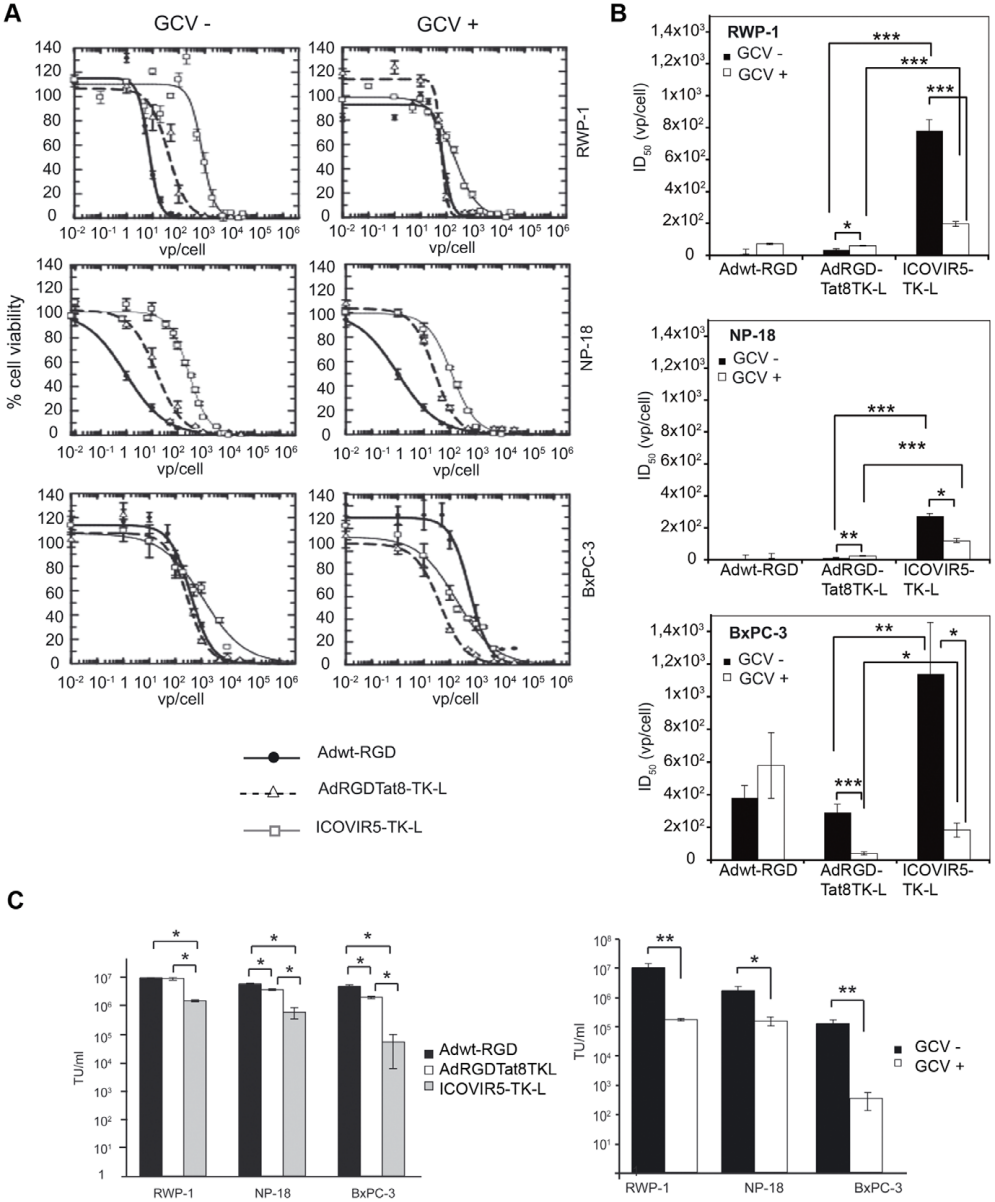
ICOVIR5-TK-L cytotoxicity *in vitro* and replication. (A) Dose-response analysis of Adwt-RGD, AdRGDTat8TK-L and ICOVIR5-TK-L in RWP-1, NP-18 and BxPC-3 cells, in presence (right) or absence (left) of 10 µg/mL GCV. Cells were incubated with the respective adenovirus during 4 hours. GCV treatment was initiated 24 hours postinfection and viability was measured by MTT 4 days postinfection. (B) ID_50_ values for each condition calculated by non-linear regression. (C) Viral replication of Adwt-RGD, AdRGDTat8TK-L and ICOVIR5-TK-L in RWP-1, NP-18 and BxPC-3 cells in the absence of GCV (left) Viral replication of ICOVIR5-TK-L in the presence or absence of GCV (right). Three ×10^4^ cells per well were infected with 10^4^ vp per cell, during 4 hours. When stated GCV treatment (10 µg/mL) was initiated 24 hours postinfection. At day 4 postinfection, cells and supernatants were harvested and the viral yield was determined by hexon protein staining. Results expressed as means of at least 3 independent experiments +/− SEM. *p<0.05, **p<0.01, ***p<0.001.

To examine the cytopathic effect of ICOVIR5-TK-L on human pancreatic adenocarcinoma cells, RWP-1, NP-18, and BxPC-3 cells were infected with increasing doses of adenovirus and cell viability was analyzed after 4 days in culture, in the presence or absence of the prodrug ganciclovir. Parental Adwt-RGD and the AdRGDTat8-TK-L were included for comparison. MTT assays showed that ICOVIR5-TK-L was able to induce cytotoxicity 3 days after culture albeit to a lesser extent than parental adenoviruses, as evidenced by a shift to the right in the dose-response curves corresponding to ICOVIR5-TK-L (Figure 2A). ID_50_ values of ICOVIR5-TK-L in all three pancreatic tumor cell lines were higher than those of the control viruses. Interestingly, in ICOVIR5-TK-L infected cells the addition of GCV significantly increased the cytotoxicity in all three cell lines, with ID_50_ values lower in the combination of ICOVIR5-TK-L/GCV than in those of ICOVIR5-TK-L treated alone This observation did not hold true for the parental adenoviruses, as the only situation in which the addition of GCV induced an increase in the cytotoxicity of the parental adenoviruses was in BxPC-3 cells treated with AdRGD-Tat8-TK-L (Figure 2B).

In order to evaluate whether the lower cytotoxicity induced by ICOVIR5-TK-L as compared to parental adenoviruses was related to a decreased ability to replicate, transducing units resulting from infected cultures of RWP-1, BxPC-3 and NP-18 cells were quantified by hexon staining four days after infection. In all three cellular contexts, ICOVIR5-TK-L infection yielded less infective particles than Adwt-RGD and AdRGD-Tat8TK-L, differences being statistically significant (Figure 2C Left).

Similarly, we decided to analyze the impact of GCV addition on viral replication by measuring viral yield in cultures of RWP-1, BxPC-3 and NP-18 cells infected with ICOVIR5-TK-L, grown in presence of absence of GCV (Figure 2C Right). A significant reduction in the quantity of infective units (up to 2-log for BxPC-3 cells) was observed for all three lines when GCV was added, similarly to what we previously described by parental AdRGDTat8TK-L [1].

### ICOVIR5-TK-L/GCV triggers tumor growth delay comparable to Adwt-RGD

**Figure 3:**
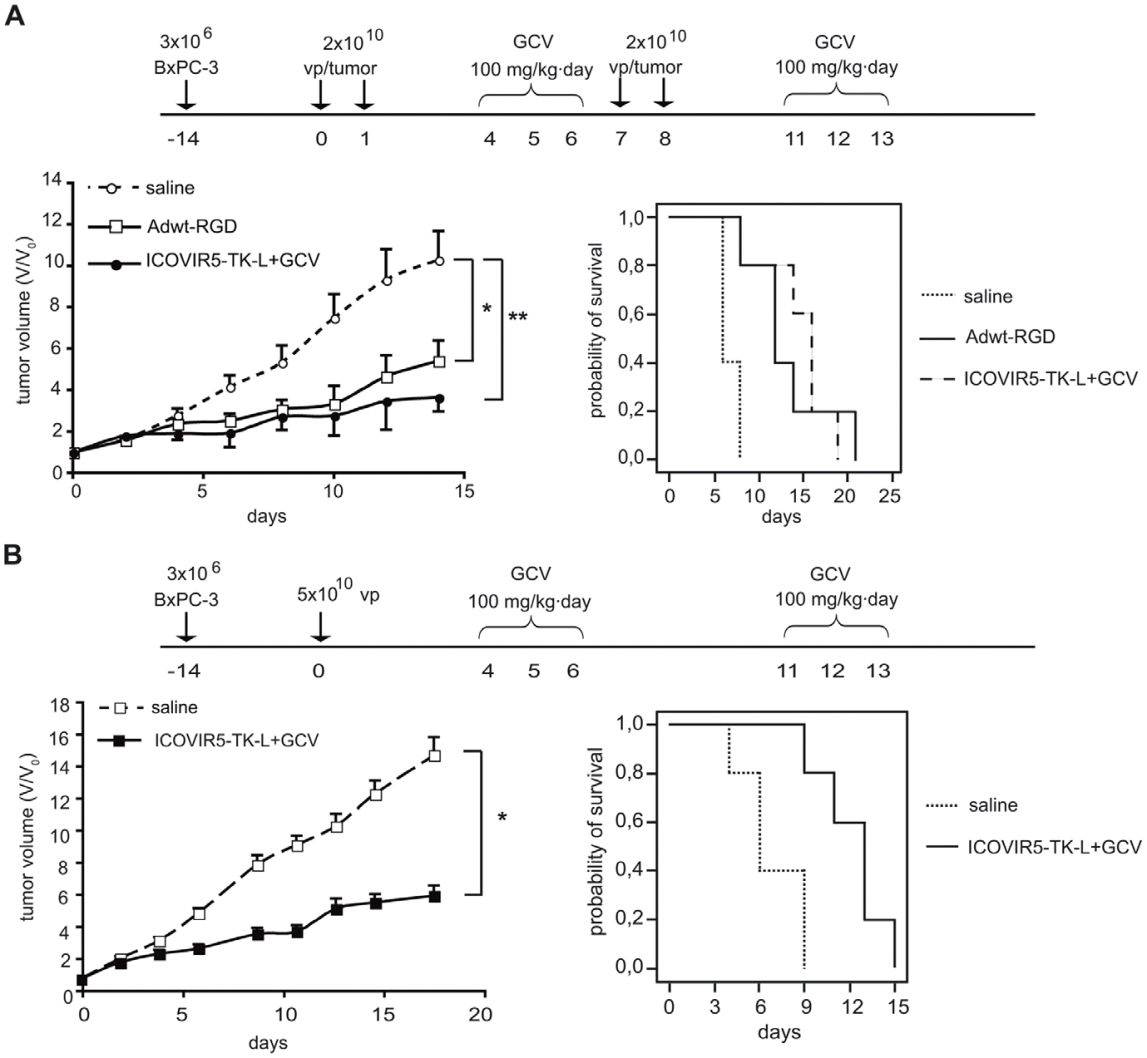
*In vivo* antitumor effect of ICOVIR5-TK-L+GCV administered locally or systemically. (A) Nude mice bearing BxPC-3 subcutaneous xenografts were treated with saline, Adwt-RGD or ICOVIR5-TK-L+GCV as indicated in the diagram. Tumor volumes were measured every other day during 2 weeks. Adwt-RGD and ICOVIR5-TK-L+GCV induced a statistically significant delay in tumor progression as compared to saline-treated controls. No significant difference was observed between Adwt-RGD and ICOVIR5TK-L+GCV treatments. Kaplan-Meier survival analysis showed a statistically significant increase in median survival of mice receiving Adwt-RGD and ICOVIR5-TK-L as compared to saline treated controls. (B) Antitumor effect of a single iv dose/mouse of 5×10^10^ vp ICOVIR5-TK-L followed by 6 doses of GCV administered as indicated in the diagram. Statistically significant tumor growth delay and increase in median survival was observed in treated animals as compared to controls. Volumes expressed as fold increase from volume at day 0 (V_0_). Results expressed as means +/− SEM (n = 5 mice/group). *p<0.05, **p<0.01.

A subcutaneous xenograft model consisting of BxPC-3 cells implanted in the flanks of nude mice was used to evaluate the antitumoral efficacy of ICOVIR5-TK-L, in comparison to an RGD-pseudotyped wild type replication-competent adenovirus Adwt-RGD. When tumors had reached an average volume of 50 mm^3^ (day = 0) mice were randomized into three groups (n = 5) receiving either a daily intratumoral dose of 2×10^10^ vp ICOVIR5-TK-L per tumor on days 0, 1, 7 and 8 followed by a daily dose of 100 mg/kg GCV intraperitoneally (i.p) on days 4, 5, 6, 11, 12 and 13; or Adwt-RGD instead of ICOVIR5-TK-L. The third group received intratumoral injections of saline instead of adenovirus. A significant delay in tumor growth was observed in the two groups receiving adenoviral treatment as compared to saline-treated control group (Figure 3A). Noteworthy, no statistically significant difference was observed between the two treated groups, suggesting that despite the fact that ICOVIR5-TK-L presents a reduced ability to replicate it can induce, when applied in combination with GCV, a similar antitumor effect as a replicative adenovirus without tumor specificity. Kaplan-Meier survival analysis showed a statistically significant survival benefit for the two adenovirus-treated groups as compared to saline group, (Log-Rank Test 0.0005 and 0.0023) but no difference was observed between the two treated groups (Figure 3A). These results demonstrate that ICOVIR5-TK-L/GCV suicide gene virotherapy has an antitumoural potency similar to that of the wild type adenovirus.

ICOVIR5-TK-L is a selective virus that replicates in cells with a pRB deficient pathway. This selectivity brings the potential to use this virus to target disseminated cancer. In order to assess whether ICOVIR5-TK-L was also able to induce growth delay when administered systemically, mice bearing BxPC-3 tumors were treated with a single intravenous injection of ICOVIR5-TK-L 5×10^10^ vp and two rounds of three consecutive doses of GCV (100 mg/Kg) at days 4,5,6 and 11,12,13 after viral injection. ICOVIR5-TK-L/GCV treatment induced a significative tumour growth inhibition, when compared to saline injected tumours (Figure 3B). Kaplan-Meier survival analysis revealed a median survival time of 6 days in control animals and of 13 days in ICOVIR5-TK-L/GCV treated mice (Log Rank Test = 0.0084) (Figure 3B).

### ICOVIR5-TK-L are detected by PET imaging in injected and contra-lateral tumors

First, we conducted a series of experiments with the recombinant adenovirus AdTK and AdTat8TK to demonstrate the detectability of the nucleoside analogue ^18^F-FEAU upon phosphorylation by the TK enzyme. ^18^F-FEAU is an optimal radiotracer to monitor TK activity as it has been reported to present high uptake rate and low background activity [24]. To this end, NP-18 pancreatic tumor cells were transduced with increasing AdTK or AdTat8TK viral doses and 24 h later incubated with 33.34 MBq of ^18^F-FEAU for 2 h. A similar viral dose dependent effect was observed by quantification of cellular radioactivity (Figure S1A). In vivo ^18^F-FEAU tumor retention was first monitored in s.c tumors derived from CWR-CLT cells stably expressing the TK gene (Figure S1B). In another set of experiments, 2×10^10^vp of recombinant AdTK were intratumorally injected into BxPC-3 xenografts. MicroPET imaging with ^18^F-FEAU was performed 5 days later. Accumulation of entrapped radiotracer was detected both in the injected tumor and in the liver, most probably as a consequence of the blood flow and the strong liver tropism of adenovirus (Figure S1C).

**Figure 4:**
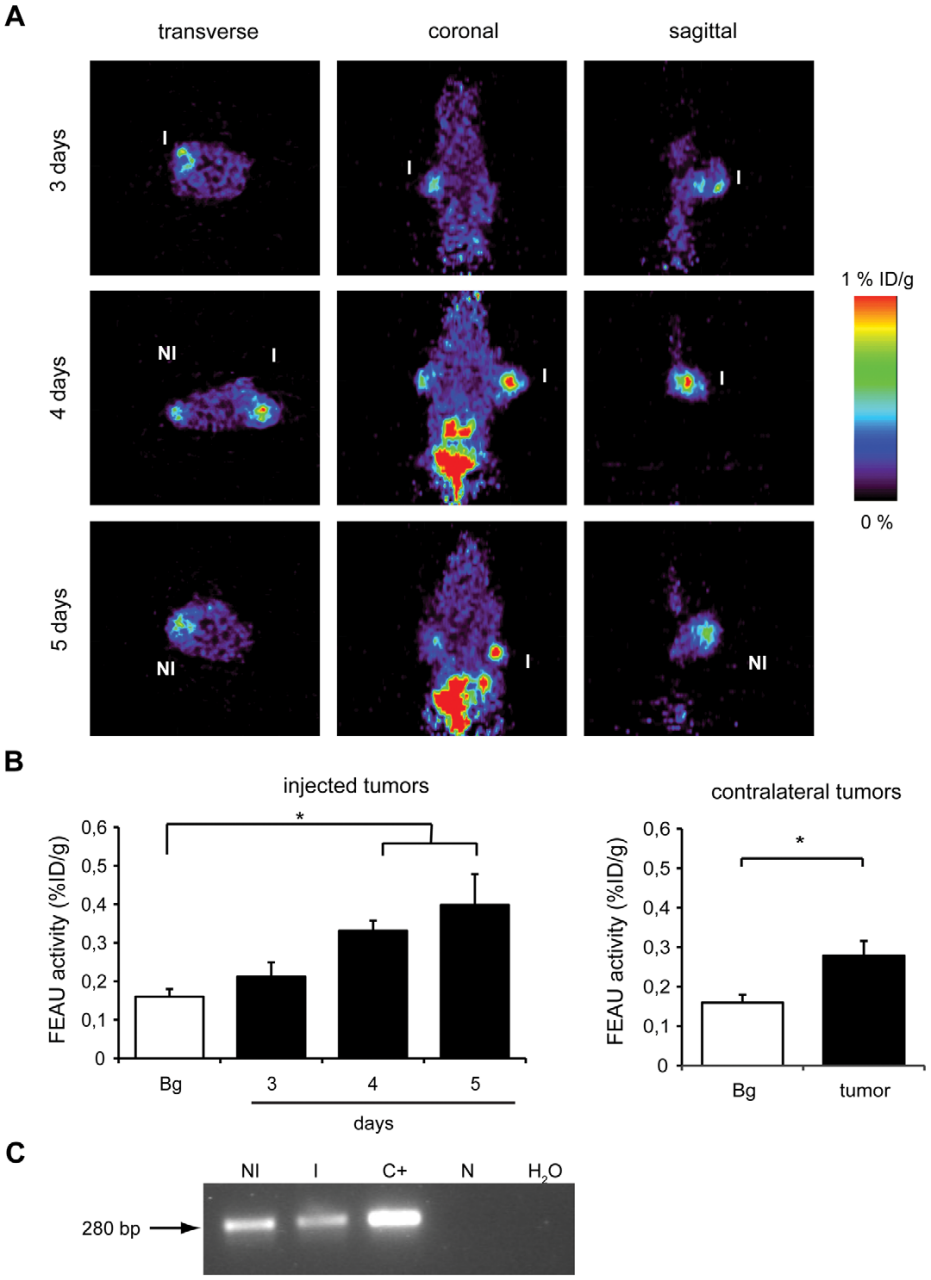
*In vivo* detection of TK activity using ^18^F-FEAU as a PET radiotracer. (A) 2-D slices showing an increased retention of radiotracer in injected (I) and in non-injected (NI) contralateral tumors. (B) Volumes of Interest (VOI) corresponding to tumoral masses were defined and ^18^F-FEAU activity was measured. At days 4 and 5 activity was higher than background signal measured in the toracic cavity, being the difference statistically significant, both in injected and also in non-injected contralateral tumors. (C) Presence of ICOVIR5-TK-L in non-injected tumors was confirmed by detection of the viral hexon gene, in tumoral tissue. N: tumor from a non-injected naïve mouse. C+: positive control of PCR reaction. *p<0.05.

To study the feasibility of detecting the presence of the oncolytic ICOVIR5-TK-L non-invasively in *vivo* by PET scans, BxPC-3 xenografts were generated in the anterior flanks of nude mice. Once the tumors reached 200 mm^3^ in size, 2×10^10^vp of ICOVIR5-TK-L were injected into the right-flank tumor. ^18^F-FEAU was i.v. injected 2 h previous to image acquisition. Images obtained at 3, 4 and 5 days after viral injection showed tumor specific detection. Interestingly, both injected and contra-lateral non-injected tumors were positive for the radiotracer. High levels of radioactivity were also observed in the gallbladder and kidneys, as renal clearance was the major route of elimination for the radiotracer (Figure 4A). Quantification of ^18^F-FEAU activity in the tumor areas revealed a significant increase in radioactive counts in the injected tumors both at 4 and 5 days after injection and although at lower levels ^18^F-FEAU activity was also significantly increased in the contra-lateral tumors (Figure 4B). Viral presence in the contra-lateral tumor was confirmed by detection of the hexon viral gene in tumor DNA extracts (Figure 4C).

### TK activity from PET scans correlates with the antitumoral effects in the different regimens of ICOVIR5-TK-L/GCV treatment

**Figure 5:**
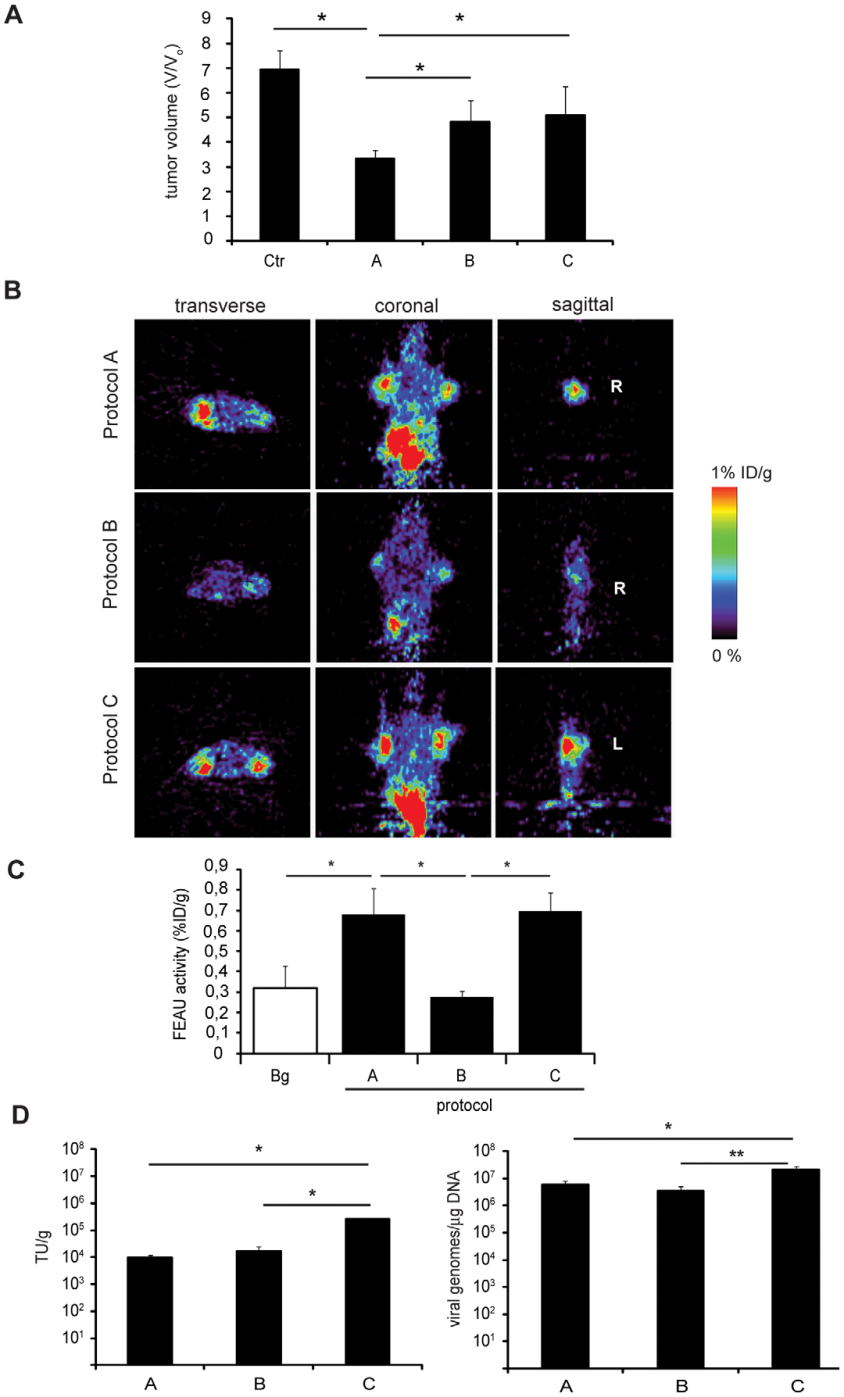
Effects of administration regimen on antitumor activity, TK activity and viral replication. (A) BxPC-3 subcutaneous xenografts were generated in nude mice that were randomized in three groups, receiving different treatments. Group A received 4 doses of 2×10^10^ vp ICOVIR5-TK-L on days 0, 1, 7 and 8, and 6 ip doses of 100 mg/kg GCV on days 4, 5, 6, 11, 12 and 13. Group B received 4 doses of 2×10^10^ vp ICOVIR5-TK-L on days 0, 1, 2, 3 and 6 ip doses of 100 mg/kg GCV on days 6, 7, 8, 9, 10 and 11. Group C received ICOVIR5TK-L as in Group A, and 6 ip injections of saline instead of GCV. A fourth group receiving saline intratumorally and ip was used as a control. Tumor volumes were measured and expressed as fold increase at day 14 from day 0. (B) PET images at day 14 showing specific retention of radiotracer in tumors. (R) Right tumor, (L) Left tumor. (C) Quantification of TK activity in tumors. GCV treatment induced a decrease in ^18^F-FEAU retention when administered as in Group B, but not as in group A, when compared to the group receiving only adenovirus. (D) Quantification of ICOVIR5-TK-L transducing units in injected tumors. Xenografts were excised at day 14 and homogenized in isotonic buffer. Extracts were applied to A549 cells and the number of infectious particles in the extracts were quantified by Hexon staining. GCV treatment produced a significant decrease in the number of ICOVIR5-TK-L transducing units regardless of the protocol applied (A or B) (left panel). (D) Q-PCR analysis of viral genomes in tumors. A reduction in viral genomes was also observed in those tumors that were treated with GCV (right panel). *p<0.05, **p<0.01.

We have previously shown that the combination protocol of a replicative competent adenovirus and GCV can display different outputs in terms of its antitumoral effects [1]. In the current work we have investigated the antitumor efficacy of ICOVIR5-TK-L and its combination with GCV under different regimens. BxPC-3 xenografts were intratumorally injected with four doses of 2×10^10^ vp of ICOVIR5-TK-L. Mice were randomized in three treated groups. Group A received two consecutive viral doses followed by three doses of GCV in two cycles. Group B received four consecutive viral doses followed by 6 doses of GCV. Group C received the four viral doses in two cycles, similarly to group A but without GCV. An effective antitumoral effect was observed in the group treated with protocol A that received virus and GCV in two cycles. Two weeks after treatment tumor size in control mice was 2.3-fold higher than tumors from treatment A (p<0.05). Furthermore, treatment A was more effective than treatments B and C (p<0.05) (Figure 5A). Interestingly quantification of TK activity, as measured by ^18^F-FEAU retention in PET scans of injected tumors revealed that treatment A and C had similar values significantly superior to those of treatment B, suggesting that GCV could be interfering in viral replication in the B protocol (Figures 5B, 5C). In fact tumor viral titers were slightly reduced when GCV was applied. However this was observed in the two conditions of GCV administration, protocols A and B (Figure 5D). The apparent discrepancy of elevated TK activity and reduced viral titers, might partially result from the 24 h delay observed between TK expression and viral release in infected cells (Figure S2), indicating that there is a population of cells that do express TK but are not yet producing viruses. Such cells, at higher GCV concentrations, as can be expected in protocol B would be rapidly eliminated resulting in reduced TK activity, whereas at lower GCV, as expected in protocol A they might survive.

Overall these results show that the combined effects of armed TK oncolytic ICOVIR5-TK-L and GCV can provide with improved antitumoral effects under an appropriate administration schedule.

## Discussion

Oncolytic adenoviruses have shown antitumoral effects and proven to be relatively safe in the clinics [25]. However, tumor response is suboptimal highlighting the need of enhanced potent oncolytic adenoviruses. Moreover, reliable methods that can trace viral replication and distribution in multiple organs are absolutely necessary to correlate vector design and dose schedule with specificity of infection and antineoplastic efficacy.

We have previously demonstrated that the combined therapy of adenoviral replication and TK/GCV cytotoxicity triggers enhanced antitumoral effects that either therapy alone under a defined regimen of virus and GCV [1]. Here we report that GCV increases the cellular killing effect of a pRB pathway-based selective adenovirus armed with the TK gene (ICOVIR5-TK-L) inducing a similar antitumor response of a highly non-selective replicating adenoviruses and shows its potential to act at distant tumor areas. Our data suggests that GCV can boost the anti-cancer effects of armed adenovirus in the context of attenuated viral oncolysis. In fact, ICOVIR5TK-L viruses showed both reduced lytic activity and viral production when compared to the replication-competent AdRGDTat8TK-L, probably related to the larger size of ICOVIR5-TK-L genome. In this direction it is well known that Ad5 capacity to carry exogenous DNA is restricted and the insertion of transgenes into the adenovirus genome can limit the oncolytic potency of the virus [26]. Interestingly, GCV improved the oncolytic capacity of ICOVIR5-TK-L in spite of interfering with viral replication, triggering similar cell death and antitumor effects in s.c xenografts as wild type adenoviruses. These suggest that a lower viral load of ICOVIR5-TK-L generated in infected cells might prove as beneficial as Adwt-RGD when GCV is administered. This might provide enhanced safety to the oncolytic ICOVIR5-TK-L virus by minimizing potential adverse events associated with viral replication in non-desired tissues.

Furthermore, our results demonstrate that viral activity can be monitored in a preclinical experimental model of pancreatic cancer by non-invasive PET imaging. We could trace TK activity of the replication-competent ICOVIR5-TK-L intratumorally injected in BxPC-3 xenografts at doses of 2×10^10^ vp. To our knowledge this is the first demonstration of monitoring a TK replication competent adenovirus in a tumor. Previous data in hepatocellular carcinoma was reported in recombinant TK adenovirus in primates and in humans [10], [12], [27]. The biochemical attributes of high uptake rate and selectivity of the ^18^F-FEAU substrate could certainly contribute to facilitate monitoring TK expression *in vivo* [28]. This high sensitivity by the PET substrate allowed for the detection of TK activity in the liver after intratumoral injection of a non-replicative TK expressing adenovirus. This viral leakness could probably result from bloodstream spreading of the virus in this pancreatic tumor model, in which s.c. xenografts are highly vascularized. This might imply that intratumoral injections of a TK therapeutic virus in this model could act at distant metastasis, or at least at liver tumoral focus, an organ where pancreatic tumors frequently metastasize.

Non-invasive imaging of a replication competent adenovirus has been nicely shown in prostate cancer and in colorectal cancer cells by monitoring the sodium iodide symporter (NIS) by SPECT imaging [29], [30], [31]. Thus, both technologies TK/PET and NIS/SPECT can be effectively used to monitor replication competent adenovirus in the body. Interestingly, TK monitoring in a replicative adenovirus administration followed by GCV treatment could also provide information on the treatment response, as we observed in the current work an association of the TK activity and the administration regimen of virus plus GCV with the antitumor effect.

An advantage of oncolytic suicide gene therapy with TK-armed oncolytic adenoviruses is that the toxic products produced within the tumor can reach cells that have not been infected, by the so-called bystander effect increasing tumor cell destruction. Despite GCV reducing total viral load the TK/GCV effects compensate on the reduced lytic activity and the final outcome is beneficial.

Although a considerable delay on tumor progression was achieved by ICOVIR5-TK-L+GCV, combination treatment failed to induce complete erradication of pre-established BxPC-3 tumors; and although it doubled mean survival, long-term survival of treated mice was not achieved. This might be related to the suboptimal spreading of viral particles within the tumor mass in rapidly growing tumors as are BxPC-3 xenografts. The redesign of adenoviruses incorporating mutations with replication enhancement capacity or engineering adenoviruses that could act on the tumor stroma could facilitate intratumoral viral spread and potentially increase long-term survival. To improve the in vivo antitumoral effect of the proposed treatment, the combination with strategies that can prime an effective anti-tumor immune response is especially appealing. Immunotherapy and cytotoxic combinations can become more specific and more effective. In fact, in the context of an immunocompetent environment, the oncolytic suicide TK/GCV therapy could potentially act as an enhancer of immunogene therapy. In this direction it has recently been shown that the administration of a recombinant adenovirus AdTK along with ganciclovir in immunocompetent mice bearing tumors, plus a chemotherapy regimen of cisplatin and gemcitabine induced marked and significant regression, similar to that achieved with the injection of Ad-INF-α or Ad-E7, an adenovirus expressing the E7 protein of the human papillomavirus, under the same chemotherapy combined treatment. This major effect was demonstrated to be due to a boost effect that chemotherapy would trigger after being primed with AdTK/GCV [32]. Although we did not directly evaluate the immunological response to ICOVIR-5TK-L, due to the low permissibility of murine cells to Ad5 replication, there are several reports showing that AdTK/GCV *in vivo* causes cell death directly and through induction of an antitumoral or antiviral immune response [33], [34]. Consistent with these data ICOVIR5-TK-L/GCV, through its ability to replicate in the tumors might enhance the vaccine effect and an appropriate GCV regimen will provide with the chemotherapeutic effect. We could then speculate that treating patients with ICOVIR5TK-L/GCV could have similar effects to a combined approach of immunotherapy and chemotherapy with the advantage of avoiding the systemic toxicity commonly associated with chemotherapy.

Treatment-related toxicity is an important consideration when developing a new cancer therapy. The safety of replication-competent adenovirus mediated suicide therapy has been evaluated in Phase I/II trials of prostate cancer and shown low toxicity [30]. Importantly, ICOVIR5-TK-L/GCV might even have reduced toxicity. Although the side effects of GCV could not be avoided, those seem to be restricted to the hematologic lineages with mild lymphopenia being the most prevalent. Viral toxicity is expected to be minimal thanks to the high level of tumor selectivity of ICOVIR5-TK-L. The different genetic modifications present in the viral backbone designed to avoid E1A expression and subsequent virus replication in normal cells are a guarantee of safety [5].

In summary, our results indicate that the expression of the TK gene in an oncolytic adenovirus can be monitored *in vivo* by PET imaging allowing for the traceability of the virus in the body. Moreover, the oncolytic suicide gene therapy based on the TK/GCV system triggers considerable antitumoral effects under an appropriate regimen. Therefore the application of ICOVIR5TK-L/GCV in pancreatic tumors holds the potential to be transferable to patients.

## Supporting Information

Figure S1 **Analysis of TK activity by ^18^F-FEAU substrate.** (A) NP-18 pancreatic cancer cells were transduced at the indicated viral doses. Twenty-four hours later cells were incubated with 0,91 µCi of ^18^F-FEAU for 2 h and radioactivity was quantified in the cell extracts. (B) Tumors from CWR-CLT cells stably expressing the TK gene were s.c. injected into the flanks of nude mice.^18^F-FEAU tumor retention was analyzed as described in materials and methods. Images show specific signal in the tumor. (C) Mice bearing BxPC-3 xenografts received three intratumoral injections of 2×10^10^ vp/tumor AdTK. Five days later ^18^F-FEAU retention was analyzed. Images show TK activity in the tumor and strong signal in the liver.
(TIF)

Figure S2 **Virus release and TK expression.** HEK 293 cells were seeded at 2×10^5^ cells per well in triplicate in 24-well plates and cultured overnight. The next day, cells were infected with ICOVIR5-TK-L at 1×10^3^ vp per cell. Four hours later, infection medium was removed and cells were washed three-times with PBS and incubated with fresh medium. (A) At the indicated time points a fraction of the supernatant was harvested and viral yield was determined by the anti-hexon staining method. Detection of viral particles became evident at 48 h post-infection. (B) RT-PCR analysis was performed to determine TK and E1A expression in RNA extracted from cell pellets at the indicated time-points. E1A and TK were first detected at 6 h and 24 h post-infection respectively.
(TIF)
