# Supplementary figures and images for: Oncolytic Adenoviruses Armed with Thymidine Kinase Can Be Traced by PET Imaging and Show Potent Antitumoural Effects by Ganciclovir Dosing
Source: PLoS One. 2011 Oct 18;6(10):e26142. doi: 10.1371/journal.pone.0026142 (PMC3196510; doi:10.1371/journal.pone.0026142)

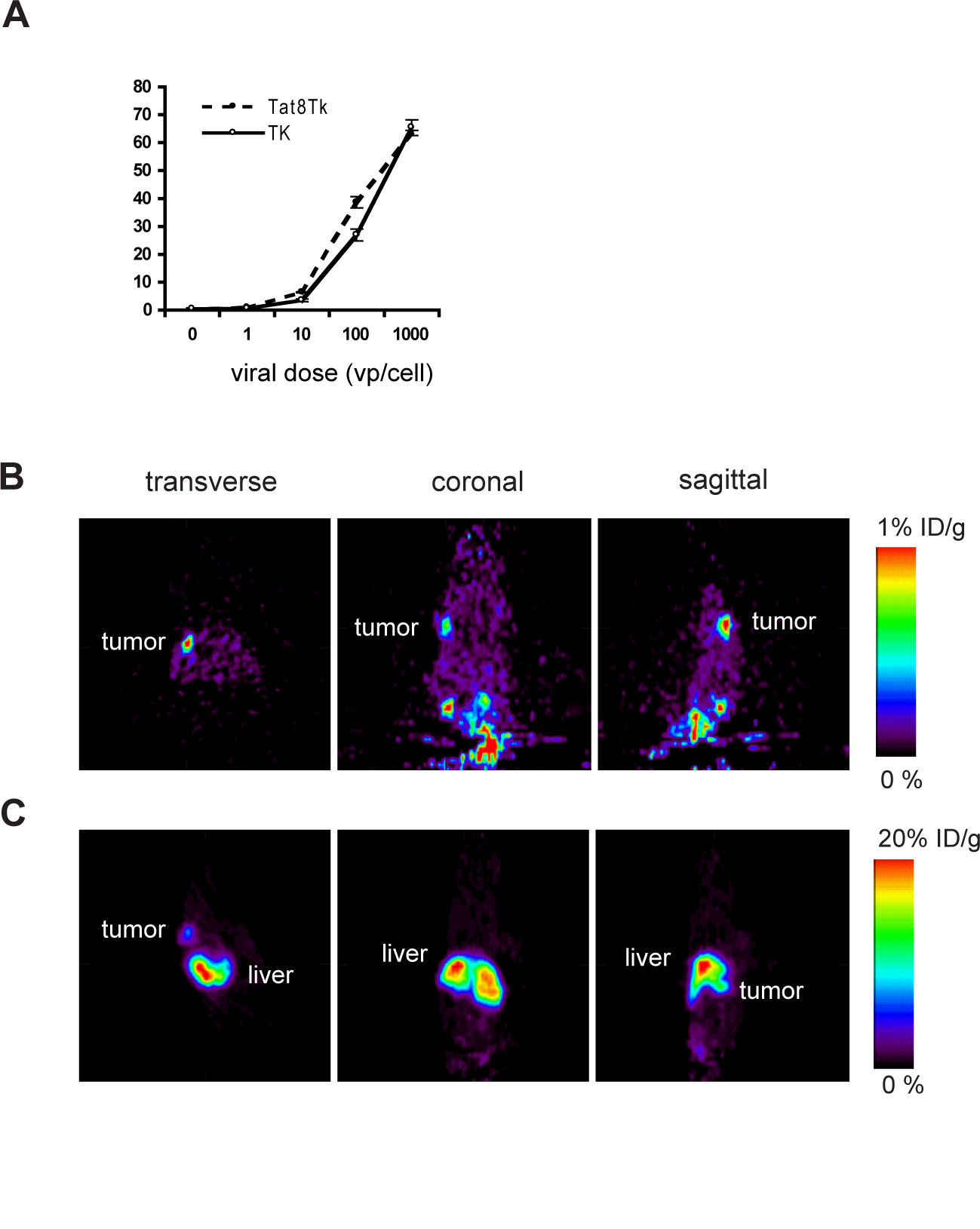

Supplement: Figure S1 — Analysis of TK activity by 18F-FEAU substrate. (A) NP-18 pancreatic cancer cells were transduced at the indicated viral doses. Twenty-four hours later cells were incubated with 0,91 µCi of 18F-FEAU for 2 h and radioactivity was quantified in the cell extracts. (B) Tumors from CWR-CLT cells stably expressing the TK gene were s.c. injected into the flanks of nude mice.18F-FEAU tumor retention was analyzed as described in materials and methods. Images show specific signal in the tumor. (C) Mice bearing BxPC-3 xenografts received three intratumoral injections of 2×10^10^ vp/tumor AdTK. Five days later 18F-FEAU retention was analyzed. Images show TK activity in the tumor and strong signal in the liver. (TIF) [file pone.0026142.s001.tif]

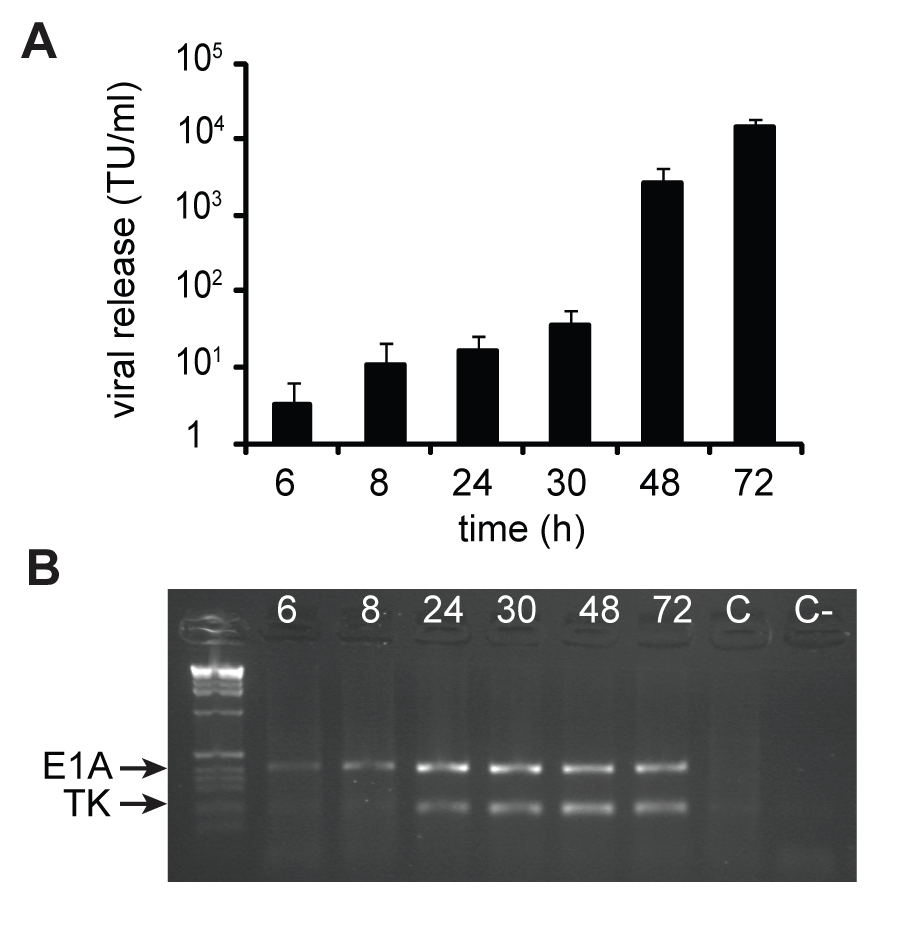

Supplement: Figure S2 — Virus release and TK expression. HEK 293 cells were seeded at 2×10^5^ cells per well in triplicate in 24-well plates and cultured overnight. The next day, cells were infected with ICOVIR5-TK-L at 1×10^3^ vp per cell. Four hours later, infection medium was removed and cells were washed three-times with PBS and incubated with fresh medium. (A) At the indicated time points a fraction of the supernatant was harvested and viral yield was determined by the anti-hexon staining method. Detection of viral particles became evident at 48 h post-infection. (B) RT-PCR analysis was performed to determine TK and E1A expression in RNA extracted from cell pellets at the indicated time-points. E1A and TK were first detected at 6 h and 24 h post-infection respectively. (TIF) [file pone.0026142.s002.tif]
